# Unveiling convergent and divergent intrinsic brain network alternations in depressed adolescents engaged in non‐suicidal self‐injurious behaviors with and without suicide attempts

**DOI:** 10.1111/cns.14684

**Published:** 2024-05-13

**Authors:** Linling Li, Zhen Liang, Guohua Li, Hong Xu, Xing Yang, Xia Liu, Xin Zhang, Jianhong Wang, Zhiguo Zhang, Yongjie Zhou

**Affiliations:** ^1^ Guangdong Provincial Key Laboratory of Biomedical Measurements and Ultrasound Imaging, School of Biomedical Engineering, International Health Science Innovation Center, Shenzhen University Medical School Shenzhen University Shenzhen China; ^2^ Department of Psychiatric Rehabilitation Shenzhen Mental Health Center/Shenzhen Kangning Hospital Shenzhen China; ^3^ Department of Computer Science and Technology, Harbin Institute of Technology Peng Cheng Laboratory Shenzhen China

**Keywords:** adolescence, depression, machine learning, non‐suicidal self‐injury, resting‐state functional connectivity, suicidal attempt

## Abstract

**Aims:**

Limited understanding exists regarding the neurobiological mechanisms underlying non‐suicidal self‐injury (NSSI) and suicide attempts (SA) in depressed adolescents. The maturation of brain network is crucial during adolescence, yet the abnormal alternations in depressed adolescents with NSSI or NSSI+SA remain poorly understood.

**Methods:**

Resting‐state functional magnetic resonance imaging data were collected from 114 depressed adolescents, classified into three groups: clinical control (non‐self‐harm), NSSI only, and NSSI+SA based on self‐harm history. The alternations of resting‐state functional connectivity (RSFC) were identified through support vector machine‐based classification.

**Results:**

Convergent alterations in NSSI and NSSI+SA predominantly centered on the inter‐network RSFC between the Limbic network and the three core neurocognitive networks (SalVAttn, Control, and Default networks). Divergent alterations in the NSSI+SA group primarily focused on the Visual, Limbic, and Subcortical networks. Additionally, the severity of depressive symptoms only showed a significant correlation with altered RSFCs between Limbic and DorsAttn or Visual networks, strengthening the fact that increased depression severity alone does not fully explain observed FC alternations in the NSSI+SA group.

**Conclusion:**

Convergent alterations suggest a shared neurobiological mechanism along the self‐destructiveness continuum. Divergent alterations may indicate biomarkers differentiating risk for SA, informing neurobiologically guided interventions.

## Graphical abstract

The maturation of network connectivity is crucial during adolescence. This study examined resting‐state alternations in depressed adolescents with or without NSSI and SA. Convergent alterations suggest a shared neurobiological mechanism along the self‐destructiveness continuum. Divergent alterations indicate biomarkers differentiating the risk for SA, informing neurobiologically guided interventions.

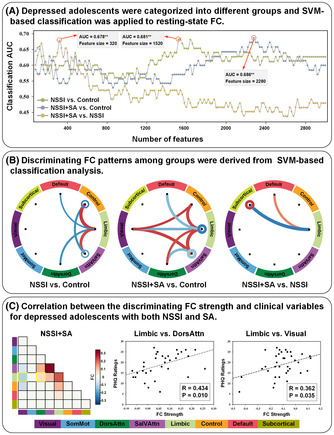

## 1 INTRODUCTION

Depression, non‐suicidal self‐injury (NSSI), and suicidal thoughts and behaviors (STBs) frequently co‐occur during adolescence, a period marked by substantial physiological, psychological, and social changes. NSSI involves deliberate, non‐lethal self‐inflicted harm lacking social sanction.^1^ Initially, adolescents may experience depression and subsequently develop NSSI as a maladaptive coping mechanism to alleviate emotional pain.^2^ The prevalence rates of NSSI are estimated at approximately 17% in community samples^3^ and exceed 40% in clinical samples.^4^ A high frequency of NSSI incidents is associated with an increased risk of suicide attempts (SAs).^5^ Adolescents engaging in repeated NSSI, coupled with a history of SA, often exhibit greater psychopathology severity compared to those with NSSI alone.^6^ Given the challenging treatment courses and elevated mortality risk associated with SA,^7^ it is crucial to understand why certain adolescents only engage in NSSI while others progress to SA. Investigating the neurobiological underpinnings of these behaviors can inform early intervention and prevention strategies promoting healthy neurodevelopmental trajectories.^8^

Investigators employed various functional magnetic resonance imaging (fMRI) experimental paradigms, revealing blunted striatal activation related to STBs and NSSI, as well as reduced frontolimbic connectivity in suicide ideators and attempters.^9^, ^10^ While these task‐specific connectivity findings are crucial for beginning to elucidate the brain basis, these studies utilized disparate emotion‐processing tasks, yielding discrepant findings. This highlights the need for understanding task‐independent brain alternations (i.e., intrinsic neural networks) associated with NSSI and SA. Resting‐state fMRI analysis has emerged as a valuable tool for investigating intrinsic large‐scale functional networks in clinical conditions. Patterns derived from task‐independent resting‐state fMRI studies are relatively stable and exhibit strong test re‐test reliability.^11^ To date, a limited number of resting‐state fMRI studies have explored NSSI and STBs in adolescents and reported reduced resting‐state functional connectivity (RSFC) between the default mode network and salience network in attempters, along with alternations of frontolimbic system for NSSI.^9^, ^12^ Moreover, many existing studies in this area are hypothesis‐driven and primarily focused on selected regions of interest (ROIs), such as the amygdala^13^ and posterior cingulate cortex.^14^ A comprehensive whole‐brain analysis is necessary to facilitate a more complete understanding of neurobiological underpinnings associated with NSSI and SA in adolescents. A recent study employed a data‐driven methodology (e.g., independent component analysis) and discovered that both STBs and NSSI were associated with brain networks linked to challenges in self‐referential processing and difficulties in future planning. Notably, NSSI specifically correlated with brain networks associated with disruptions in interoceptive awareness.^15^ However, this study utilized an overlapping sample of depressed adolescents, making distinctions based on their history of NSSI or STBs without considering the co‐occurrence of both behaviors.

Investigating the clinical phenomena of NSSI and suicidal behavior poses challenges due to their independent and co‐occurring nature in adolescence.^16^ The proposed Gateway Theory suggests that NSSI precedes the development of suicidal behaviors, acting as a gateway from self‐injury to more extreme forms of self‐injury with similar experiential qualities (i.e., SAs).^17^ As approximately a third of adolescents with suicidal thoughts eventually progress to SAs,^18^ and NSSI behavior plays a specific role in the transition from thoughts to actions,^19^ there is a pressing need for studies exploring neurobiological correlates capable of differentiating between adolescents engaged in NSSI who attempt suicide and those who do not. Such studies have the potential to enhance our current knowledge and inform clinical practice. There may be a complex neurobiological interaction and overlap in RSFC patterns between NSSI and SA. However, thus far, few researchers have utilized resting‐state fMRI to investigate the similarities and differences in intrinsic brain networks between NSSI and NSSI+SA in depressed adolescent populations.

In this study, we examined the history of SA among depressed adolescents with NSSI, aiming to reveal both convergent and divergent patterns of self‐injury behaviors (NSSI only, NSS + SA) compared to clinical controls. To derive meaningful insights, our findings were based on a comparison between diagnostic controls and individuals with the same mental disorder alongside NSSI or SA. This approach is particularly valuable as it is more likely to capture the specific effects of these behaviors within the context of that particular disorder. Whole‐brain RSFC alternations were extracted using data‐driven support vector classification to overcome the multiple‐comparison problems of their multivariate nature.^20^ Based on the existing literature, we anticipate that convergent and divergent intrinsic brain network alternations in this study would also center around the Limbic network, along with other brain networks associated with emotional regulation and cognitive control. A comprehensive exploration of the neural correlates underlying NSSI and SA may contribute to a more thorough understanding of the nature of the link between these two types of self‐injury behavior in adolescents.

## 2 METHODS

### 2.1 Participants

In this study, 114 adolescents were recruited from the Department of Depression at Shenzhen Kangning Hospital. The protocol for this study was approved by the Research Ethics Committee of Shenzhen Kangning Hospital (No: 2020‐K021‐04‐1). Informed and written consent was obtained from both the participants themselves and their parents or caregivers. The Chinese version of the Functional Assessment of Self‐Mutilation (FASM)^21^ was used to assess self‐injury behaviors. Patients were categorized into three groups: (1) Control group, depressed adolescents without NSSI and SA; (2) NSSI group, depressed adolescents with NSSI but no SA; (3) NSSI+SA group, depressed adolescents with a history of both NSSI and SA. More details can be found in Appendix S1: Section 1.1.

### 2.2 Data acquisition and preprocessing

All MRI scans were acquired at the Department of Radiology at Shenzhen Kangning Hospital with a 3 T scanner (Prisma, Siemens, Germany). Participants completed a resting‐state fMRI scan (TR of 2000 ms, slice thickness of 2.5 mm, 58 slices) with eyes closed and a high‐resolution T1‐weighted MRI scan (slice thickness of 1 mm, 144 slices). All fMRI data were preprocessed using Data Processing Assistant for Resting‐State fMRI (DPARSFA) based on SPM12. More details can be found in Appendix S1: Section 1.2.

### 2.3 Functional network construction

Whole‐brain RSFC matrices were calculated based on Pearson's correlation in eight distinct brain networks given by the Schaefer cortical atlas with 200 parcels^22^ and Melbourne Subcortical Atlas with 32 parcels^23^ (Appendix S1: Section 1.3 and Figure S1). The feature vector of RSFC was constructed by vectorizing the upper triangular part of the correlation matrix of each subject.

### 2.4 Support vector machine training and classification

In this study, the identification of differential RSFC between groups was achieved through the application of a support vector machine (SVM) classification. Considering the relatively high dimensionality of whole‐brain brain network features, the use of machine learning approaches becomes crucial in addressing the multiple‐comparison problem inherent in such multivariate data.^20^, ^24^, ^25^ The original multiclass problem was translated into a series of binary comparisons,^26^ and linear SVM classifiers^27^ were trained on RSFC data. The F‐scores method^28^ was applied for feature selection to remove redundant information and to avoid overfitting.^29^ The F‐score of each feature is calculated by the ratio between the variance between groups and the variances within each of the two groups. The larger the *F*‐score is, the more likely the feature is to be more discriminative between the two groups.

A nested leave‐one‐out‐cross‐validation (LOOCV) strategy was implemented to mitigate overfitting and provide a more accurate estimate of the classifier's performance. Before analysis, each feature was normalized across subjects in the training sample by subtracting the mean and dividing by the standard deviation, and the same mean and standard deviation were used to scale the test data. The feature number was tested from 20 to 3000 with a step of 20. For each LOOCV iteration, the dataset (N subjects of two groups in total) was divided into a test set (one subject) and a training set (N‐1 subjects). The F‐scores of all features were calculated and then ranked within the training set. Specificity, sensitivity, and area under curve (AUC) were calculated to measure the performance of the selected classifier. The prediction performance was calculated for all the LOOCV iterations of each step of feature size, and the smallest step that achieved the highest prediction AUC value was chosen.

### 2.5 Evaluating contributions to a predictive model

Since a linear kernel SVM was used, we could extract the classification weights as a measure of differences between groups. Consensus features were defined as the common features always selected to form the final feature set for each LOOCV iteration. The weights of the consensus features were the average value of the classification weight across all iterations of LOOCV (normalized to [−1 1], as shown in Figure S2). Then, we applied a two‐step filtering process on the consensus features to extract features with the most significant discriminative power. First, we extracted the consensus features with weights greater than the mean plus the standard deviation. Then for the retained features, we calculated the intra‐ and inter‐network mean classification weights and percentages. Specifically, the mean weights were calculated by averaging the weights of all the features within each network and between pairs of networks. The percentages of consensus features occurring within and between different networks were as the number of selected connections divided by the number of all possible connections. Subsequently, we only retain features whose distribution percentage values are greater than the mean plus the standard deviation. The final remaining set of features is referred to as discriminating features (as shown in Figure S3). The predictive contributions of discriminating features were also visualized at the nodal level. Specifically, the classification weight of each region was calculated by summing one‐half of the weights associated with that region.^30^ Subsequently, brain regions that play a more important role in the classification can be identified.

### 2.6 Statistical analysis

All statistical analyses were performed using SPSS (IBM, Armonk, NY). The chi‐square test and one‐way analysis of variance (ANOVA) test were performed to analyze the group differences in the demographic data. *p* < 0.05 was considered statistically significant. The statistical significance of selected classifiers was evaluated by the non‐parametric permutation tests (5000 times). For NSSI and NSSI+SA groups, we extracted the mean intra‐ and inter‐network discriminating RSFC strength (compared with Control and a direct comparison between NSSI and NSSI+SA), and the partial Pearson's correlation analyses (age and gender were included as the confounding factors) were performed between these RSFC and clinical parameters.

## 3 RESULTS

### 3.1 Demographics and clinical characteristics

As shown in Table 1, no significant group differences were observed in age, gender, education, and self‐reported severity of depression and anxiety symptoms. In the NSSI+SA group, the proportion of patients diagnosed for the first time is significantly higher than in the other two groups. Concerning the descriptive characteristics of NSSI, the NSSI+SA scored higher on the NSSI frequency and attention seeking of NSSI function.

**TABLE 1:** Clinical characteristics.

Item	Control	NSSI	NSSI + SA	*p*‐Value
	*N* = 34	*N* = 39	*N* = 36	
Gender (M/F)	9/25	6/33	5/31	0.332^a^
Age (years)	15.65 ± 1.61	15.38 ± 1.63	15.06 ± 2.08	0.385^b^
Edu (years)	10.06 ± 1.70	9.82 ± 1.78	9.44 ± 2.42	0.430^b^
PHQ scores	15.53 ± 6.33	17.64 ± 5.88	18.94 ± 5.51	0.056^b^
GAD scores	11.41 ± 5.76	12.79 ± 5.26	13.06 ± 5.72	0.419^b^
First diagnosed (%)	88%	82%	56%	0.001^a^
Depression course (month)	10.38 ± 11.18	12.86 ± 17.26	13.03 ± 10.97	─
NSSI frequency (last year)	─	12.00 ± 9.41	8.00 ± 6.02	0.034^c^ ^,^ *
NSSI function (emotion regulation)	─	1.62 ± 0.78	1.82 ± 0.87	0.295^c^
NSSI function (attention seeking)	─	0.41 ± 0.55	0.75 ± 0.67	0.018^c^ ^,^ *
NSSI function (social avoidance)	─	0.80 ± 0.72	0.90 ± 0.85	0.577^c^
Lifetime suicide attempts	─	─	5.53 ± 10.86	─
ERQ‐CA reappraisal	17.68 ± 5.19	15.59 ± 4.16	16.39 ± 4.96	0.178^b^
ERQ‐CA suppression	13.35 ± 3.04	14.31 ± 2.35	14.92 ± 3.11	0.073^b^
Current psychopharmacotherapy	0	1 (2.6%)	1 (2.8%)	0.393^a^
Head movements (FD Jenkinson)	0.068 ± 0.023	0.073 ± 0.029	0.075 ± 0.031	0.599^b^

*Note*: Values represent mean (SD) or *n* (%) unless otherwise indicated.

Abbreviations: Depression course, calculated without first diagnosed patients; ERQ‐CA, Emotion Regulation Questionnaire for Children and Adolescents; GAD, Generalized Anxiety Disorder; NSSI, non‐suicidal self‐injury; PHQ, Patient Health Questionnaire.

^a^ Chi‐square test.

^b^ One‐way ANOVA.

^c^ Two‐sample *t*‐test.

* *p* < 0.05.

### 3.2 Classification performance based on RSFC

**FIGURE 1:**
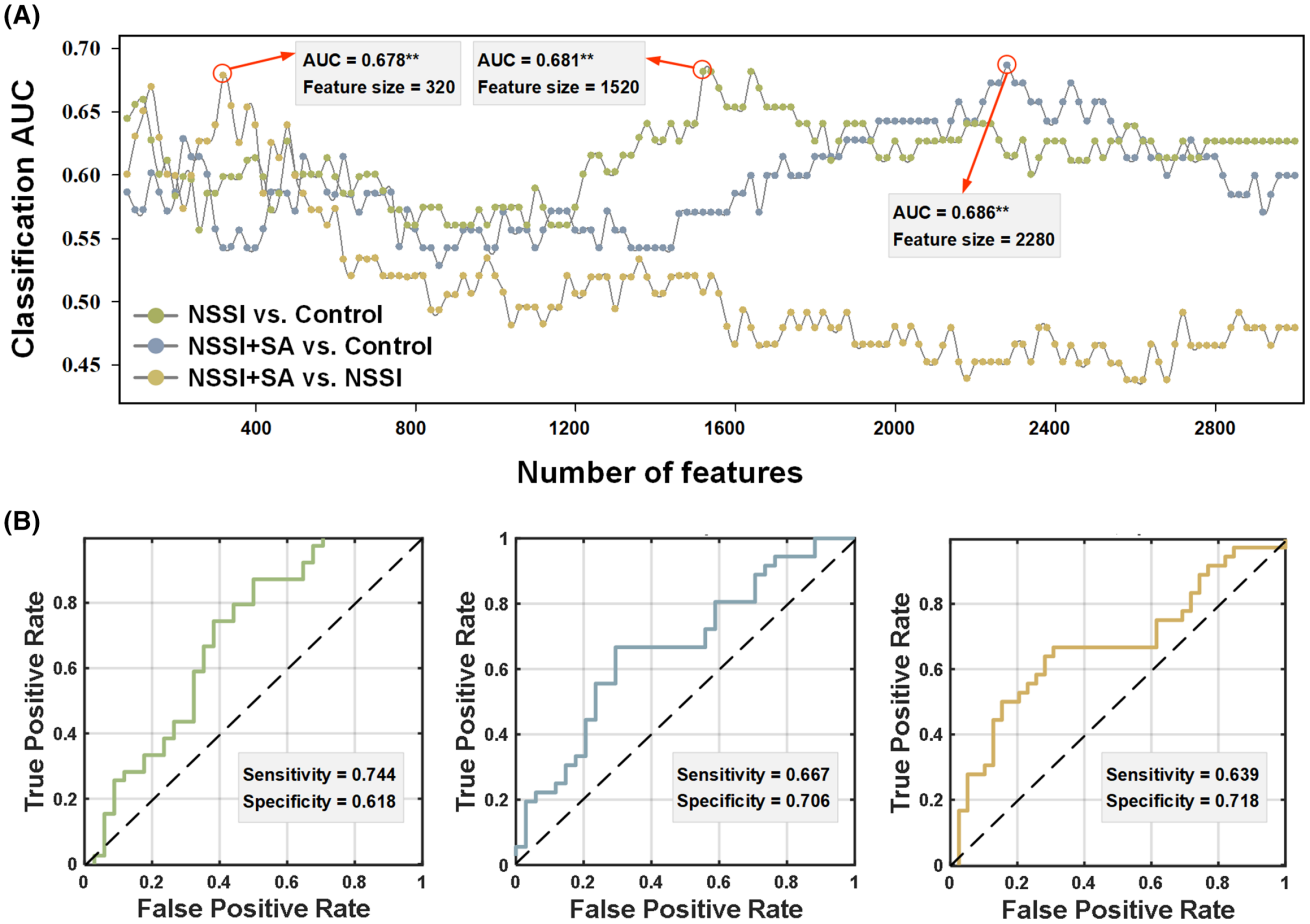
Classification performance. (A) Each curve corresponds to the classification between different groups, and the maximum value was selected (**p < 0.005). (B) The corresponding ROC curves.

Patients were categorized into different groups using linear SVM classifiers based on RSFC. Feature sets of varying sizes were employed, and the optimal size, yielding the highest classification performances, was determined (as illustrated in Figure 1). These findings underscore the discriminative capability of RSFC in distinguishing between various patient groups.

### 3.3 Discriminating RSFCs between groups

**FIGURE 2:**
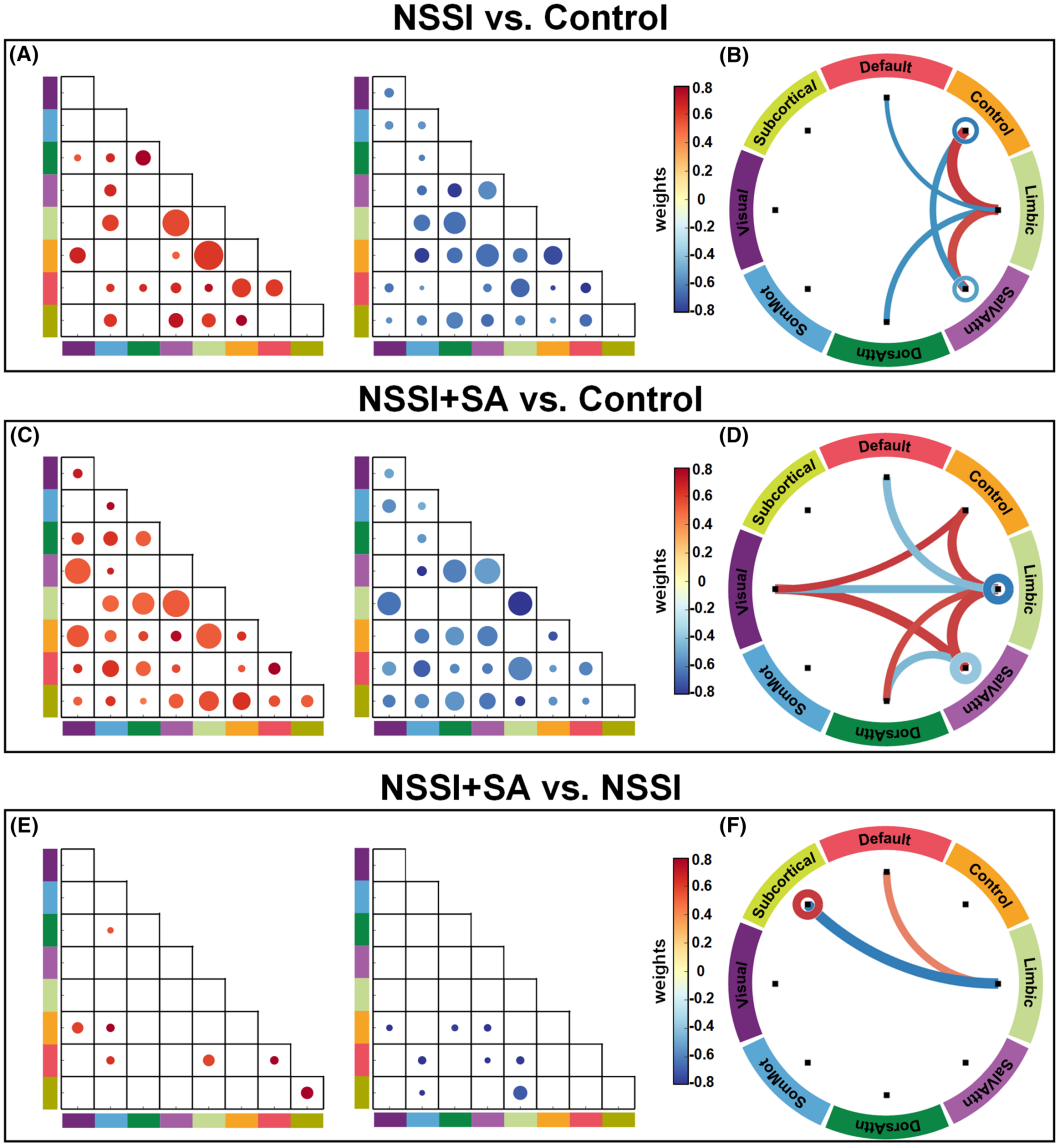
Group differences in RSFC were identified through SVM‐based classification. To identify RSFC features with the most significant discriminative power, a two‐step filtering process was applied to the consensus features derived from SVM classification, resulting in the final set of discriminating features. Matrices on the left (A, C, and E) show the classification weights of consensus features selected after the first‐step filtering by applying a threshold of mean plus the standard deviation on the weights. The size of the dots in the matrices is in proportion to the intra‐ and inter‐network mean weights, with a consistent scale of enlargement across all matrices. Circular graphs on the right (B, D, and F) illustrate the network‐level architecture of final discriminating features selected after the second‐step filtering by applying a threshold of mean plus the standard deviation on the percentages of features within and between different networks. Dots (in the matrices) and lines (in the circular graphs) with warm color represent positive discriminating weights and the reverse for dots or lines with cold color. The line thickness is in proportion to the percentage of discriminating features.

Group differences in RSFC were identified through SVM‐based classification (as shown in Figure 2). Compared to the Control group, the NSSI group exhibited increased inter‐network RSFCs of the Limbic network (with the Control and SalVAtten networks), decreased intra‐network RSFCs of the Control and SalVAttn networks, and decreased inter‐network RSFCs of the Limbic network (with the Default and DorsAttn networks), as well as between the Control and SalVAttn networks. When compared with the Control group, the NSSI+SA group showed convergent alternations similar to the NSSI group, including inter‐network RSFCs of the Limbic network (with the Default, Control, and SalVAtten networks) and intra‐network RSFCs of the SalVAttn network. The NSSI+SA group also exhibited additional alternations in inter‐network RSFCs of the Visual network (with the Control, Limbic, and SalVAttn networks) derived from the comparison with the Control group, and inter‐network RSFCs of the Limbic network (with the Subcortical and Default networks), as well as intra‐network RSFCs of the Subcortical network, derived from the comparison with the NSSI group.

**FIGURE 3:**
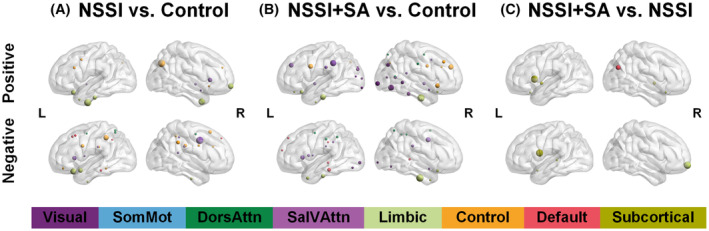
Brain regions contributed more to the classification between the NSSI and Control (left panel), between the NSSI+SA and Control (middle panel), and between the NSSI+SA and NSSI (right panel). The colors of nodes represent the different brain networks, and the node size is in proportion to the classification weight of each region.

The 3D glass brain representations in Figure 3 (listed in the Table S1) illustrate the nodal‐level representation of discriminating feature weights between groups. When comparing the NSSI group with the Control group, brain regions with larger positive discriminating weights are predominantly concentrated in the Limbic (e.g., orbital frontal cortex and temporal pole) and Control (e.g., prefrontal cortex and precuneus) networks, while brain regions with larger negative weights are present in the Limbic (e.g., temporal pole), Control (e.g., cingulate and supramarginal gyrus), and SalVAttn (e.g., insular and precentral gyrus) networks. When comparing the NSSI+SA group with the Control group, brain regions with larger positive weights are located in the Limbic (e.g., orbital frontal cortex and temporal pole), Control (cingulate and prefrontal cortex), SalVAttn (e.g., prefrontal and inferior parietal cortex), and Visual networks, while brain regions with larger negative weights are located in the Limbic (e.g., temporal pole) and SalVAttn (insular cortex and parietal operculum) networks. When comparing the NSSI+SA group with the NSSI group, brain regions with larger positive weights are present in the Default (e.g., posterior parietal cortex) and Subcortical (e.g., caudate) networks, while brain regions with larger negative weights are present in the Limbic (e.g., orbitofrontal cortex) and Subcortical (e.g., caudate) networks. Based on the distribution of discriminating weights in brain regions, it can be observed that convergent alterations in the NSSI group and NSSI+SA group are primarily concentrated in the Limbic network. As for the divergent alterations in the NSSI+SA group, they are mainly focused on the Visual, Limbic, and Subcortical networks.

### 3.4 Correlation between discriminating RSFCs and clinical variables

**FIGURE 4:**
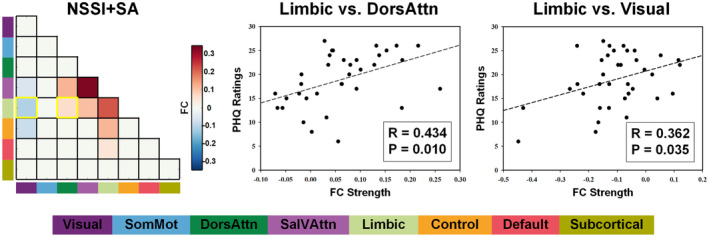
Correlation between the discriminating features of RSFC and severity of depressive symptoms in the NSSI+SA group.

Based on the discriminating features extracted from the comparison between the NSSI+SA group and Control group, the RSFCs between the Limbic and DorAttn networks (*R* = 0.434, *p* = 0.010) and RSFCs between the Limbic and Visual networks (*R* = 0.362, *p* = 0.035) in the NSSI+SA group showed a significant positive correlation with PHQ scores (Figure 4). No significant correlation was observed between the discriminating features and any of the clinical parameters for the NSSI group.

## 4 DISCUSSION

Existing evidence suggests that the predisposition to suicidal and non‐suicidal behaviors in adolescents, at least in part, is mediated by neurobiological factors. The altered cerebral function might play a key biological role in NSSI and SA among depressed adolescents.^9^, ^10^, ^12^ This study is the first to examine both convergent and divergent neurobiological alternations underlying adolescent NSSI with or without a history of SA. Based on our findings, convergent alterations in NSSI and NSSI+SA are predominantly centered on the inter‐network RSFC between the Limbic network and the three core neurocognitive networks (SalVAttn, Control, and Default networks). Additionally, divergent alterations observed in the NSSI+SA group primarily focus on the Visual, Limbic, and Subcortical networks. Moreover, the severity of depressive symptoms in the NSSI+SA group is significantly correlated only with certain discriminating inter‐network RSFCs of the Limbic network. This reinforces the notion that these observed alternations could not all be solely explained by increased depression severity. We discuss potential interpretations of these findings below.

The relationship between NSSI and SA is complicated, and both lie along a spectrum of self‐destructive actions in adolescence.^17^ When studying the neural mechanisms underlying these behaviors, our focus on networks is crucial because network maturation is a central feature of brain development during adolescence.^31^ Studies on normative development have demonstrated that functional brain networks increasingly segregate throughout adolescence,^32^ making it a vulnerable period for emotional problems.^33^ Importantly, the networks supporting these processes continue to mature in adolescence^31^ and are aberrant in depressed adolescents.^34^ NSSI is a key risk factor for future SAs, with some of the same neurobiological systems implicated in both NSSI and SA. Initial findings support the view that NSSI and SA in adolescents are characterized by abnormal RSFCs of specific brain regions; however, there is a lack of characterization regarding alternations in the overall brain network patterns.^9^, ^12^

Menon et al. proposed a triple‐network model (SalVAttn, Control, and Default networks) for neuropsychiatric disorders, and these three networks are associated with emotional regulation and cognitive processing.^35^ According to our results, convergent alternations for NSSI and NSSI+SA primarily focus on the inter‐network RSFC between the Limbic network and the three core neurocognitive networks. The Limbic network comprises two main nodes, namely the orbitofrontal cortex (OFC) and temporal pole.^36^ The OFC plays a pivotal role in integrating emotional information and is associated with emotional regulation in emotional disorders. In NSSI participants, greater activation was observed in the OFC during emotional processing tasks.^37^, ^38^, ^39^ As part of an extended Limbic system, the temporal pole contributes to both social and emotional processes, and dysfunction in the temporal pole has been observed in clinical disorders involving socioemotional regulation.^40^ The SalVAttn network, primarily centered in the anterior insular and cingulate cortex, is involved in emotion‐processing and monitoring salient events.^36^ Increased sensitivity to socioaffective pain contributes to the onset of self‐injury behaviors in adolescents, and it is largely believed that it is a product of an imbalance between elevated response to affective information in the environment by the SalVAttn network,^41^, ^42^ and blunted regulation by the Control network associated with effortful emotion regulation and cognitive control.^43^ The Default network is associated with internally oriented attention.^44^ Therefore, because self‐injury behaviors represent a maladaptive strategy that stems from greater difficulty with socioemotional awareness and regulation,^45^ specific patterns of network dysfunction between the Limbic and core neurocognitive networks may underlie these behaviors. These observations emphasize theories proposing a potential continuum of self‐destructiveness^46^ and indicate a potential neurobiological underpinning of the continuum from self‐injury to suicidal behavior.

Furthermore, it has been suggested that individuals engaging in both NSSI and SA may present with more complex psychopathology.^47^ In addition to the aforementioned convergent alternations with the NSSI group, divergent alternations were observed for the NSSI+SA group, involving the Visual, Limbic, and Subcortical networks, further highlighting the complexity of their neural connectivity patterns. In the context of NSSI, adolescents show difficulties with social cue interpretation, deficits in interpersonal problem‐solving, and a tendency to negatively interpret social situations.^48^ Consistent findings revealed that adolescents with repeated NSSI, who additionally reported a history of SAs, presented greater severity of psychopathology, such as a lower level of functioning, compared to those with NSSI only.^6^ During the maintenance of NSSI, heightened negative bias may contribute to the development of SA.^47^ Hence, our findings of alternated RSFCs associated with the Visual network in the NSSI+SA group could be interpreted from an “information processing” perspective.^49^, ^50^ Vision is an important part of the selective attention process, and the dorsal Visual network is associated with spatial awareness and guidance of actions.^51^ Hence, the processing bias in adolescents with NSSI+SA may be initiated as a perceptual visual bias and then eventually lead to a series of cognitive and affective symptoms.

In the direct comparison between the NSSI group and the NSSI+SA group, both cohorts reported emotion regulation as their primary motivation for NSSI behaviors, and the NSSI+SA group reported a higher rating for the attention‐seeking factor. The heightened need for attention seeking in adolescents with SA was consistent with Joiner's “interpersonal‐psychological theory of suicidal behavior,” which states belongingness as one of the main predictors of suicidal behavior.^46^ Emotion regulation was the most common reason for NSSI consistently suggested in many previous studies.^52^ While a basic understanding of the relationship between NSSI and emotion regulation deficits has been established, a recent study supports the possibility of unique associations between emotion regulation deficits with more frequent NSSI engagement and more SAs.^53^ According to our results, the NSSI+SA group reported a higher frequency of self‐injury behaviors, which means that suicidal behavior may gradually evolve into another coping strategy due to the heightened habituation to pain and fear resulting from repeated self‐injury behaviors when emotion dysregulation persists. Besides, differences in RSFC between the NSSI and the NSSI+SA group were primarily associated with the basal ganglia of the Subcortical network. Traditionally assigned roles within the motor domain, recent research suggests that the basal ganglia have connections with a broad Limbic network and contribute to various Limbic and cognitive processes, including emotion recognition, reward‐, and decision‐making.^54^ Thus, our study extends the understanding of the link between emotional regulation deficits and suicidal behaviors by revealing differences in RSFC related to the Subcortical and Limbic networks. As the self‐reported questionnaire for emotion regulation did not yield significant differences among groups, future studies could employ a more objective assessment of emotion regulation function to validate the results observed here.

There are several limitations to consider in this study. First, the cross‐sectional design limits our ability to establish the causal relationship or determine the development trajectory of the observed aberrant patterns. To fully comprehend the emergence and progression of these patterns, longitudinal studies that track individuals over time are needed. Second, the reliance on retrospective self‐report for collecting data on NSSI, SAs, and depression symptoms introduces the potential for reporting or recall biases, which may impact the accuracy and reliability of the obtained information. Future research could consider incorporating more objective measures to mitigate these biases. Third, the number of patients was limited, and the correlation analysis results did not survive correction for multiple comparisons. The classification performance, though significantly surpassing the chance level, is around 70%. Our aim here is not to build a reliable classifier for disease diagnosis but to identify differential RSFCs between groups. Future studies could increase sample size, involve multiple research centers, and incorporate different data analysis methods to build a reliable classifier for disease diagnosis.^55^ Last but not least, the proportion of patients diagnosed for the first time differs among the three groups, and there are a higher number of females in all three groups. To gain a more comprehensive understanding, future studies should investigate these neural circuits regarding potential sex differences and different psychiatric conditions.

## 5 CONCLUSIONS

This study represents the first attempt to explore the convergent and divergent neurobiological alternations associated with NSSI and SA in adolescents. By focusing on network‐level analysis, this study expands our current understanding of the neural mechanisms underlying these behaviors at a circuit level. The results imply the existence of a continuum of self‐destructiveness in adolescents and provide insights into the neural mechanism contributing to this continuum. Additionally, the findings have the potential to assist in identifying individuals at risk for SA among those involved in NSSI, thereby contributing to the development of neurobiologically informed interventions.

## AUTHOR CONTRIBUTIONS

Conception and design of this study: Y.Z. and Z.Z.; Acquisition of data: G.L., H.X., X.Y., X.L., and X.Z.; Analysis of data: L.L. and Z.L.; Drafting of the article: L.L.; Critical revision of the article: J.W., Y.Z., and Z.Z.; All authors reviewed the manuscript.

## FUNDING INFORMATION

This work was supported by the National Natural Science Foundation of China [No. 62276169 and 82272114], the Natural Science Foundation of Guangdong Province, China [No. 2023A1515010840], the Shenzhen Science and Technology Program [No. 2022SHIBS0003, RKX20220705152815035, KCXFZ20201221173613036, SZSM202011014, and SZGSP013], the Medical‐Engineering Interdisciplinary Research Foundation of Shenzhen University, and the Shenzhen University‐Lingnan University Joint Research Programme.

## CONFLICT OF INTEREST STATEMENT

The authors declare no conflicts of interest.

## Supporting information


Appendix S1.

## Data Availability

The data and code used to support the findings of this study are available from the corresponding author upon request.
