# Supplementary material for: Unveiling convergent and divergent intrinsic brain network alternations in depressed adolescents engaged in non‐suicidal self‐injurious behaviors with and without suicide attempts
Source: CNS Neurosci Ther. 2024 May 13;30(5):e14684. doi: 10.1111/cns.14684 (PMC11090151; doi:10.1111/cns.14684)
Supplement: Supplementary file 1 — Appendix S1. [file CNS-30-e14684-s001.docx]

**Unveiling convergent and divergent intrinsic brain network alternations in depressed adolescents engaged in non-suicidal self-injurious behaviors with and without suicide attempts**

Linling Li^1^, Zhen Liang^1^, Guohua Li^2^, Hong Xu^2^, Xing Yang^2^, Xia Liu^2^, Xin Zhang^2^, Jianhong Wang^2^, Zhiguo Zhang^3*^, Yongjie Zhou^2*^

^1^ Guangdong Provincial Key Laboratory of Biomedical Measurements and Ultrasound Imaging, School of Biomedical Engineering, International Health Science Innovation Center, Shenzhen University Medical School, Shenzhen University, Shenzhen, China

^2^ Department of Psychiatric Rehabilitation, Shenzhen Mental Health Center/Shenzhen Kangning Hospital, Shenzhen, China .

^3^ Department of Computer Science and Technology, Harbin Institute of Technology, Peng Cheng Laboratory, Shenzhen, China

**Correspondence:** Zhiguo Zhang, Department of Computer Science and Technology, Harbin Institute of Technology, HIT Campus of University Town of Shenzhen, NanShan District, Shenzhen, 518055, China. Email: zhiguozhang@hit.edu.cn. Yongjie Zhou, Department of Psychiatric Rehabilitation, Shenzhen Mental Heath Center/Shenzhen Kangning Hospital, 77 Zhenbi Road, Pingshan District, Shenzhen, 518003, China. Email: qingzhu1108@126.com.

**Supplementary materials**

**1. Methods**

## **1.1. Participants**

Depressed adolescents were recruited from the Department of Depression at Shenzhen Kangning Hospital. Inclusion criteria were as follows: (1) age 12 to 18 years, (2) diagnosed with current depression by senior psychiatrists according to the Diagnostic Criteria and Statistical Manual of Mental Disorder Fifth Edition (DSM-5). The exclusion criterion consisted of a history of any serious neurological or intellectual disability, contraindications to MRI scanning, and meeting for a lifetime or current criteria for Mania, Psychosis, or any substance use disorders.

The Chinese version of the Functional Assessment of Self-Mutilation (FASM) [1] was used to assess methods, frequency, and functions of NSSI behaviors in the past year. Participants were asked if they had engaged in any of the 10 listed self-harm behaviors in the past 12 months. Participants who answered “yes” were NSSI+ while those who answered “no” were NSSI-. The reported frequency of NSSI behaviors in the last year was converted to ratings on a 5-point scale (0: never; 1: 0-25%; 2: 25%-50%; 3: 50%-75%; 4: >75%). Besides, participants were asked if they had tried to take their life before. Participants who answered “no” were SA-. Participants who answered “yes” were further asked to provide more details, including their suicidal thoughts, methods employed for suicidal attempts, timing of occurrences, and the consequences. In the end, specialized psychiatrists used this information to decide which participants truly qualified as having had suicide attempts, and they were subsequently classified as SA+. Similar assessment methods have been employed in previous studies [2-4].

The Chinese version of the FASM uses a three-factor model, named emotion regulation, attention seeking, and social avoidance, which gives rise to a reliable structure of the NSSI function in the Chinese cultural context. Scores for items belonging to each factor were summed to obtain the scores for each factor. To make the endorsement of the three factors comparable, the scores were scaled by dividing the summed scores by the number of subscales for each factor. Considering the well-established link between emotion regulation deficits and self-injury behavior, all the enrolled adolescents completed the Chinese version of ERQ-CA [5] to assess individual differences in the habitual use of two emotion regulation strategies.

## **1.2. Data acquisition and preprocessing**

MRI scanning was performed at the Department of Radiology at Shenzhen Kangning Hospital using a 3.0-Tesla scanner MRI (Prisma, Siemens, Germany). Resting-state fMRI was collected using an echo-planar imaging (EPI) sequence with the following parameters: TR/TE = 2000/30 ms; flip angle = 90⁰; slice thickness = 2.5 mm; acquisition matrix = 88×88; number of slices = 58; and 240 time points. During the scanning, each participant was asked to keep still with their eyes closed, and not to think about anything or fall asleep. High-resolution three-dimensional brain volume T1-weighted imaging was collected using the following parameters: TR/TE/IT = 2530/2.27/1100 ms; flip angle = 7⁰; slice thickness = 1 mm; acquisition matrix = 256×256; number of slices = 144. The preprocessing of resting-state fMRI was performed using Data Processing Assistant for Resting-State fMRI Advanced Edition V2.2 (DPARSFA, http://rfmri.org/DPARSF) toolkit with Statistical Parametric Mapping (SPM8, https://www.fil.ion.ucl.ac.uk/spm/) in MATLAB (R2010b) (MathWorks, Natick, MA, USA).

The main preprocessing steps were as follows: (1) The first 5 volumes were removed to allow for signal equilibration; (2) slice-timing correction was performed and the middle slice was used as the reference slice; (3) motion correction realignment was performed; five participants with excessive head motion, as operationalized by having a mean FD_Jenkinson_>0.2mm, were excluded; (4) Spatial normalization was performed to the Montreal National Institute space (MNI) with a voxel size of 3×3×3mm^3^; (5) to reduce the effect of physiological noise, a series of nuisance covariates were regressed out using the Friston-24 model [6], including the mean signal of the white matter, cerebrospinal fluid signal, and head motion parameters; (6) temporal bandpass filtering (0.01-0.1 Hz) was further performed to reduce low-frequency drift and high-frequency noise; (7) spatial smoothing was performing using a 4 mm full-width at high maximum (FWHM) Gaussian kernel. Additionally, to ensure that residual motion was not a confound, mean framewise displacement was compared between groups, and no significant effect of motion was observed (one-way ANOVA, P = 0.599).


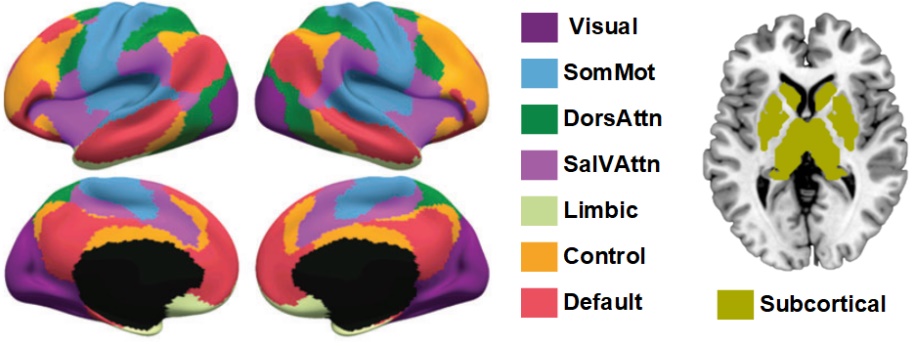


### **FIGURE S1.** Whole-brain functional parcellation.

## **1.3. Functional network construction**

To define network nodes, human brains were parcellated into 232 cortical and subcortical regions of interest (ROIs). The 200 Cortical ROIs were obtained from the scale-200 version of the whole-brain functional parcellation of Schaefer et al [7]. The 32 subcortical ROIs were obtained from a recent subcortical functional parcellation [8]. The 232 ROIs can be assigned into eight distinct brain networks: (1) visual network, (2) somatomotor network (SomMot), (3) dorsal attention network (DorsAttn), (4) salience/ventral attention network (SalVAttn), (5) limbic network, (6) control network, (7) default mode network, and (8) subcortical network [9]. We extracted the averaged BOLD time series for each of the 232 ROIs for each subject. A 232×232 correlation matrix for each subject was subsequently constructed by calculating Pearson’s correlation coefficients between the BOLD time series from all possible pairs of ROIs. A Fisher’s r-to-z transformation was further applied to the correlation matrices. The upper triangular part of the resting-state FC matrix was extracted to construct one feature vector for each subject.

**2. Results**

## **2.1. Classification results based on resting-state FC**

Resting-state fMRI FCs that provide relevant information to distinguish between each pair of groups. Matrices in FIGURE S2 show the unfiltered consensus features which were normalized to [-1 1] across all connections.


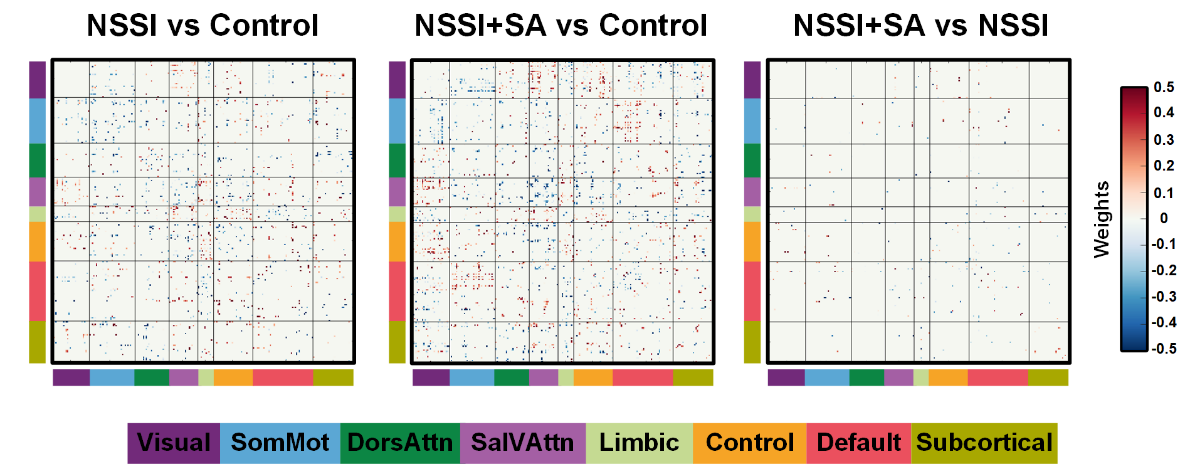


### **FIGURE S2.** The mean weights of all consensus features.

In order to extract the most discriminating features at the network level, we filtered the consensus features from two perspectives: classification weights and network-level distribution percentage. FIGURE S3 shows the mean functional connectivity of the final discriminating features for each group.


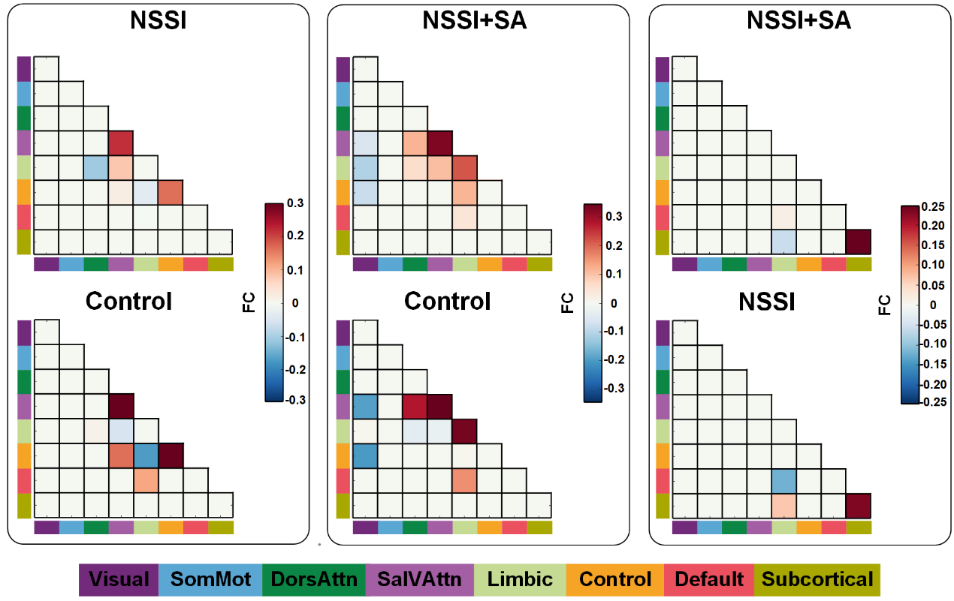


### **FIGURE S3.** The mean resting-state functional connectivity changes between groups for the discriminating features extracted from SVM classification.

The predictive contributions of the consensus intra- and inter-modular features with both greater classification weights and densities were also visualized at the nodal level. The brain regions that contributed more to the classification between groups are listed in the supplementary TABLE S1. The anatomical labels and MNI coordinates were provided.

### **TABLE S1.** Information on brain regions contributed more to the classification between groups.

| **NSSI vs. Control** | | | |  | **NSSI+SA vs. Control** | | | |
| --- | --- | --- | --- | --- | --- | --- | --- | --- |
| **ROI** | **x** | **y** | **z** |  | **ROI** | **x** | **y** | **z** |
| **Positive weights** |  |  |  |  | **Positive weights** |  |  |  |
| LH_SalVentAttn_FrOperIns_1 | -40 | -4 | -4 |  | LH_Vis_5 | -26 | -96 | -12 |
| RH_SalVentAttn_FrOperIns_1 | 40 | 6 | -16 |  | LH_Vis_9 | -22 | -96 | 6 |
| RH_SalVentAttn_FrOperIns_2 | 46 | -4 | -4 |  | LH_Vis_11 | -40 | -84 | 10 |
| RH_SalVentAttn_FrOperIns_3 | 36 | 24 | 4 |  | LH_Vis_13 | -6 | -88 | 28 |
| LH_Limbic_OFC_1 | -24 | 22 | -20 |  | LH_Vis_14 | -24 | -88 | 24 |
| LH_Limbic_TempPole_1 | -30 | -6 | -40 |  | RH_Vis_1 | 38 | -34 | -24 |
| LH_Limbic_TempPole_2 | -44 | -20 | -30 |  | RH_Vis_3 | 28 | -68 | -12 |
| LH_Limbic_TempPole_3 | -28 | 10 | -34 |  | RH_Vis_7 | 16 | -46 | -2 |
| RH_Limbic_OFC_3 | 14 | 64 | -8 |  | RH_Vis_8 | 30 | -94 | -4 |
| RH_Limbic_TempPole_1 | 30 | 8 | -38 |  | RH_Vis_9 | 8 | -76 | 8 |
| LH_Cont_PFCl_4 | -44 | 20 | 28 |  | RH_Vis_10 | 22 | -60 | 8 |
| LH_Cont_PFCl_5 | -42 | 6 | 44 |  | RH_Vis_15 | 32 | -74 | 32 |
| LH_Cont_pCun_1 | -10 | -74 | 38 |  | RH_DorsAttn_Post_2 | 52 | -60 | 10 |
| RH_Cont_PFCv_1 | 34 | 22 | -8 |  | RH_DorsAttn_Post_3 | 60 | -16 | 34 |
| RH_Cont_PFCl_5 | 30 | 48 | 28 |  | RH_DorsAttn_Post_6 | 14 | -74 | 52 |
| RH_Cont_pCun_1 | 14 | -70 | 36 |  | RH_DorsAttn_FEF_1 | 34 | -4 | 52 |
| **Negative weights** |  |  |  |  | LH_SalVentAttn_ParOper_2 | -62 | -26 | 28 |
| LH_DorsAttn_Post_8 | -30 | -60 | 60 |  | LH_SalVentAttn_ParOper_3 | -60 | -40 | 36 |
| LH_DorsAttn_Post_9 | -6 | -60 | 56 |  | LH_SalVentAttn_FrOperIns_1 | -40 | -4 | -4 |
| LH_DorsAttn_FEF_1 | -32 | -4 | 54 |  | LH_SalVentAttn_PFCl_1 | -28 | 42 | 32 |
| RH_DorsAttn_FEF_1 | 34 | -4 | 52 |  | RH_SalVentAttn_TempOccPar_2 | 60 | -38 | 16 |
| LH_SalVentAttn_ParOper_2 | -62 | -26 | 28 |  | RH_SalVentAttn_FrOperIns_1 | 40 | 6 | -16 |
| LH_SalVentAttn_FrOperIns_2 | -34 | 20 | 6 |  | RH_SalVentAttn_FrOperIns_2 | 46 | -4 | -4 |
| LH_SalVentAttn_FrOperIns_3 | -38 | 0 | 10 |  | RH_SalVentAttn_Med_1 | 8 | 10 | 42 |
| RH_SalVentAttn_TempOccPar_2 | 60 | -38 | 16 |  | LH_Limbic_OFC_1 | -24 | 22 | -20 |
| RH_SalVentAttn_TempOccPar_3 | 60 | -26 | 28 |  | LH_Limbic_OFC_2 | -10 | 36 | -20 |
| RH_SalVentAttn_PrC_1 | 50 | 4 | 40 |  | LH_Limbic_TempPole_1 | -30 | -6 | -40 |
| RH_SalVentAttn_Med_2 | 10 | -36 | 46 |  | LH_Limbic_TempPole_2 | -44 | -20 | -30 |
| LH_Limbic_OFC_1 | -24 | 22 | -20 |  | RH_Limbic_OFC_2 | 28 | 22 | -20 |
| LH_Limbic_TempPole_1 | -30 | -6 | -40 |  | RH_Limbic_TempPole_2 | 46 | -12 | -34 |
| LH_Limbic_TempPole_4 | -42 | 8 | -18 |  | RH_Limbic_TempPole_3 | 26 | -10 | -32 |
| RH_Limbic_TempPole_2 | 46 | -12 | -34 |  | LH_Cont_pCun_1 | -10 | -74 | 38 |
| LH_Cont_Par_3 | -46 | -42 | 46 |  | LH_Cont_Cing_1 | -4 | -28 | 28 |
| LH_Cont_Temp_1 | -60 | -42 | -14 |  | LH_Cont_Cing_2 | -4 | 4 | 30 |
| LH_Cont_OFC_1 | -32 | 42 | -14 |  | RH_Cont_PFCv_1 | 34 | 22 | -8 |
| LH_Cont_Cing_1 | -4 | -28 | 28 |  | RH_Cont_PFCl_5 | 30 | 48 | 28 |
| LH_Cont_Cing_2 | -4 | 4 | 30 |  | RH_Cont_PFCl_6 | 40 | 34 | 38 |
| RH_Cont_Par_1 | 62 | -38 | 36 |  | RH_Cont_pCun_1 | 14 | -70 | 36 |
| RH_Cont_Par_2 | 52 | -42 | 48 |  | RH_Cont_Cing_2 | 4 | 2 | 30 |
| RH_Cont_Cing_1 | 6 | -24 | 30 |  | RH_Cont_PFCmp_1 | 8 | 30 | 28 |
| RH_Cont_Cing_2 | 4 | 2 | 30 |  | **Negative weights** |  |  |  |
| RH_Cont_PFCmp_1 | 8 | 30 | 28 |  | LH_Vis_2 | -26 | -78 | -14 |
| RH_Cont_PFCmp_2 | 8 | 24 | 56 |  | LH_Vis_7 | -6 | -92 | -4 |
| LH_Default_PFC_11 | -40 | 18 | 50 |  | RH_Vis_3 | 28 | -68 | -12 |
| LH_Default_PFC_12 | -24 | 24 | 48 |  | RH_Vis_8 | 30 | -94 | -4 |
| LH_Default_pCunPCC_4 | -6 | -54 | 42 |  | LH_DorsAttn_Post_4 | -54 | -26 | 42 |
| LH_Default_PHC_1 | -26 | -32 | -18 |  | LH_DorsAttn_Post_6 | -32 | -48 | 46 |
| RH_Default_PFCdPFCm_5 | 16 | 46 | 44 |  | LH_DorsAttn_FEF_1 | -32 | -4 | 54 |
|  |  |  |  |  | RH_DorsAttn_Post_4 | 46 | -38 | 50 |
| **NSSI+SA vs. NSSI** | | | |  | RH_DorsAttn_Post_7 | 34 | -48 | 50 |
| **ROI** | **x** | **y** | **z** |  | RH_DorsAttn_Post_8 | 26 | -62 | 58 |
| **Positive weights** |  |  |  |  | RH_DorsAttn_FEF_2 | 26 | 8 | 58 |
| 7Networks_LH_Limbic_OFC_1 | -24 | 22 | -20 |  | LH_SalVentAttn_ParOper_2 | -62 | -26 | 28 |
| 7Networks_RH_Limbic_OFC_2 | 28 | 22 | -20 |  | LH_SalVentAttn_ParOper_3 | -60 | -40 | 36 |
| 7Networks_RH_Default_Par_1 | 46 | -70 | 28 |  | LH_SalVentAttn_FrOperIns_2 | -34 | 20 | 6 |
| aGP-rh | 18 | 0 | -2 |  | LH_SalVentAttn_FrOperIns_3 | -38 | 0 | 10 |
| aGP-lh | -16 | 0 | -2 |  | LH_SalVentAttn_FrOperIns_4 | -52 | 8 | 10 |
| aCAU-lh | -12 | 14 | 6 |  | RH_SalVentAttn_TempOccPar_3 | 60 | -26 | 28 |
| **Negative weights** |  |  |  |  | RH_SalVentAttn_PrC_1 | 50 | 4 | 40 |
| 7Networks_LH_Limbic_TempPole_4 | -42 | 8 | -18 |  | RH_SalVentAttn_FrOperIns_1 | 40 | 6 | -16 |
| 7Networks_RH_Limbic_OFC_3 | 14 | 64 | -8 |  | LH_Limbic_TempPole_2 | -44 | -20 | -30 |
| THA-DP-lh | -14 | -30 | 2 |  | LH_Limbic_TempPole_3 | -28 | 10 | -34 |
| pCAU-lh | -12 | 4 | 16 |  | RH_Limbic_OFC_2 | 28 | 22 | -20 |
|  |  |  |  |  | RH_Limbic_TempPole_1 | 30 | 8 | -38 |
|  |  |  |  |  | RH_Limbic_TempPole_2 | 46 | -12 | -34 |
|  |  |  |  |  | RH_Limbic_TempPole_3 | 26 | -10 | -32 |
|  |  |  |  |  | LH_Default_Temp_4 | -58 | -30 | -4 |
|  |  |  |  |  | LH_Default_PFC_7 | -8 | 60 | 20 |
|  |  |  |  |  | LH_Default_PFC_9 | -12 | 48 | 44 |
|  |  |  |  |  | LH_Default_pCunPCC_3 | -4 | -30 | 36 |
|  |  |  |  |  | LH_Default_PHC_1 | -26 | -32 | -18 |
|  |  |  |  |  | RH_Default_Temp_5 | 52 | -32 | 2 |

**References**

1. Qu D, Wang Y, Zhang Z, et al. Psychometric properties of the Chinese version of the functional assessment of self-mutilation (FASM) in Chinese clinical adolescents. *Front Psychiatry.* 2021;12:755857.

2. He H, Hong L, Jin W, et al. Heterogeneity of non-suicidal self-injury behavior in adolescents with depression: latent class analysis. *BMC Psychiatry.* 2023;23(1):301.

3. Sun R, Ren Y, Li X, et al. Self-compassion and family cohesion moderate the association between suicide ideation and suicide attempts in Chinese adolescents. *J Adolesc.* 2020;79:103-111.

4. Xu M, Zhang X, Li Y, et al. Identification of suicidality in patients with major depressive disorder via dynamic functional network connectivity signatures and machine learning. *Transl Psychiatry.* 2022;12(1):383.

5. Gullone E, Taffe J The emotion regulation questionnaire for children and adolescents (ERQ-CA): a psychometric evaluation. *Psychol Assess.* 2012;24(2):409-417.

6. Friston KJ, Williams S, Howard R, et al. Movement-related effects in fMRI time-series. *Magn. Reson. Med.* 1996;35(3):346-355.

7. Schaefer A, Kong R, Gordon EM, et al. Local-global parcellation of the human cerebral cortex from intrinsic functional connectivity MRI. *Cereb Cortex.* 2018;28(9):3095-3114.

8. Tian Y, Margulies DS, Breakspear M, et al. Topographic organization of the human subcortex unveiled with functional connectivity gradients. *Nat Neurosci.* 2020;23(11):1421-1432.

9. Yeo BT, Krienen FM, Sepulcre J, et al. The organization of the human cerebral cortex estimated by intrinsic functional connectivity. *J Neurophysiol.* 2011;106(3):1125-1165.
